# Mirror Therapy and Action Observation Therapy to Increase the Affected Upper Limb Functionality in Children with Hemiplegia: A Randomized Controlled Trial Protocol

**DOI:** 10.3390/ijerph18031051

**Published:** 2021-01-25

**Authors:** Rocío Palomo-Carrión, Juan Carlos Zuil-Escobar, Myriam Cabrera-Guerra, Paloma Barreda-Martínez, Carmen Belén Martínez-Cepa

**Affiliations:** 1 Department of Nursery, Physiotherapy and Occupational Therapy, Faculty of Physiotherapy and Nursery, University of Castilla-La Mancha, 45071 Toledo, Spain; Rocio.Palomo@uclm.es; 2 Department of Physiotherapy, Faculty of Medicine, CEU-San Pablo University, 28925 Madrid, Spain; mcguerra@ceu.es (M.C.-G.); paloma.barreda@ceu.es (P.B.-M.)

**Keywords:** action observation therapy, infantile hemiplegia, mirror therapy, upper extremity

## Abstract

The movements of the affected upper limb in infantile hemiplegia are slower and clumsy. This leads to a decrease in the use of the affected hand. The visual effect obtained using the mirror box and the observation of actions in another individual can activate the same structural neuronal cells responsible for the execution of these actions. This research will study the affected upper limb functionality in hemiplegia infantile from 6 to 12 years old after the application of two intervention protocols: observation action therapy and mirror therapy combined with observation action therapy. Children with a diagnose of congenital infantile hemiplegia will be recruited to participate in a randomized controlled trial with two intervention protocols during four weeks (1 h per/day; 5 sessions per/week): Mirror Therapy Action Observation (MTAO) or Action Observation Therapy (AOT). The study variables will be: spontaneous use, measured with the Assisting Hand Assessment (AHA); manual ability measured with the Jebsen Taylor Hand Function Test (JTHFT); surface electromyography of the flexors and extensors muscles of the wrist and grasp strength through a grip dynamometer. Four assessments will be performed: At baseline situation, at the end of treatment, 3 and 6 months after treatment (follow-up assessments). This study will study the effects of these therapies on the use of the affected upper limb in children with hemiplegia.

## 1. Introduction

Infantile hemiplegia is a subtype of infantile cerebral palsy (CP), characterized by the involvement of one of the halves of the body due to a brain injury. Its prevalence is one case per 1300 live births [1]. The upper limbs are affected more than the lower limbs. The affected hand has a deficit in proprioception and tactile perception, which hinders fine motor skills, generally those of the fingers and the strength exerted by them [2]. Movements in the affected upper limb are slower and clumsy and are accompanied by mirror movements (MM) which are involuntary symmetrical movements observed in the contralateral upper limb to the voluntary upper limb movements. These movements are temporally observed in the development of the healthy child and then disappear. When these movements are subsequently present, they are considered abnormal [3]. Mild MM are common in preschool children but often appear in children with hemiplegia [4]. Although the mechanisms of mirror movements are not fully understood, it is likely that they are due to abnormal organization of the development of the motor system. Thus, alterations in the maturation of the transcallosal pathways have been suggested [4,5]. Moreover, children with hemiplegia have deficits in the selective control of the affected fingers and a diminished use of them. [6]. Consequently, the use of the affected hand is reduced, which is commonly known as “developmental neglect”, affecting activities of daily living [7,8,9].

Spasticity is defined as “a sensory-motor control disorder resulting from an injury to the upper motor neuron, which presents as an involuntary intermittent or sustained activation of the muscles” [10]. The spasticity includes the velocity-dependent increase in tonic stretching reflexes (muscle tone) and phase stretching reflexes (exaggerated tendon jerk) [11], as well as flexor and extensor spasms, flexor reflexes and altered motor control. Clinically, it is often difficult to differentiate the spasticity from the symptoms and signs caused by structural changes in the muscles [10,11].

Children with spastic hemiplegia show significant restriction in their daily activities due to the limited active range of movement of the affected upper limb, specifically in the elbow, as well as the alterations in muscle activity observed by electromyography. Thus, the affected side shows more co-contractions than the non-affected side. Surface electromyography is a good evaluation system to assess the activation of the motor unit in children with hemiplegia [12,13,14]. The restriction on the child’s participation in the environment due to the non-use of the affected upper limb is caused by the lack of exteroceptive and proprioceptive information at cortical level, as a consequence of the brain injury [15,16]. Therefore, the deficits in the structure, as well as the decrease in the movement amplitude or the spasticity would not be the exclusively causes of the activity limitation [16]. This developmental restriction decreases the learning to use the affected upper limb, producing exclusive dominance of the unaffected upper limb. The child uses compensatory strategies to avoid using the affected side [16]. This non-use increases muscle tone, reduces active movement, affects the growth of the affected upper limb and reduces the strength of this arm [16,17].

Increased muscle tone due to spasticity is often reduced using antispastic treatment (botulinum toxins, phenol…) [18]. However, these treatments do not necessarily improve upper limb functionality. An improvement in the quality of movement can appear, but it would not increase the spontaneous use of the affected upper limb. Therefore, it may be necessary to use treatments that promote the function and activity-participation to improve the functionality of the affected upper limb and its performance in daily life [19].

Mirror therapy is based on visual stimulation [20]. During mirror therapy, a mirror is placed on the midsagittal plane of the person, reflecting the unaffected side as if it were the affected side [20]. Therefore, the movements of the unaffected side create the illusion of normal movements of the affected side [21]. The illusion of the mirror can prevent or reverse a learned non-use of the affected limb [22]. One of the advantages of this therapy is its use as a self-administered home therapy [21]. Bruchez et al. [23] have shown the efficacy of mirror therapy in children with hemiplegia aged 6 to 18 years, improving the quality of movement and perception of the affected upper limb through feedback visualization of the healthy limb [23]. The replacement of the visual information of the affected upper limb with a mirror reflection of the non-affected upper limb improves motor control of children with unilateral cerebral palsy or hemiplegia [24] and obstetrical brachial palsy [24]. In hemiparesis, visual feedback increases the excitability of the ipsilateral motor cortex, optimizes the congruence between afferent (visual) feedback and promotes an internal representation of motor movements [25].

The positive effects could result from the coupling between the two upper limbs. This is supported by evidence that such simultaneous movements with both affected and unaffected limbs modulate interhemispheric inhibition, allowing the unaffected hemisphere to facilitate activation of the affected hemisphere. [26]. On the other hand, it has been shown that the observation of actions in another individual can activate the same neuronal structures, responsible for the execution of these actions, known as the mirror neuron network. From here appears the AOT, which stimulates the motor behaviour of the affected upper limb through the observation of sequences of systematic activities and their subsequent execution [27,28]. Transcranial magnetic stimulation (TMS), used while the subject was observing the actions, showed an increase in the excitability of the corticospinal tract, resulting in muscle contraction patterns very similar to those observed in action, favouring motor activity. Hence, as there is a greater corticospinal activation, there could be a greater cortical representation and therefore an increase in the spontaneous use of the affected upper limb in infantile hemiplegia [28].

No literature describing research combining mirror therapy and action observation could be found. Their combined use could increase the perception of the affected upper limb and facilitate motor recruitment and quality of movement, through visual illusion. In addition, with action observation, the spontaneous use and participation of the affected upper limb in daily activities through repetition of observed tasks can be increased. The combination of mirror therapy and the action observation therapy (MTAO) could facilitate the quality of movement of the upper limb, improving its structure and function and providing greater cortical activation and spontaneous use, through the repetition of specific movements. Furthermore, it can be done at home using a single intervention protocol. Conducting the therapy in the child’s home can involve the child in repeated practice of tasks and involve the family in the treatment process [29]. It has been reported that parents of children with disabilities have higher levels of stress than parents of typically developing children [30], which may suggest that the home is a viable setting for implementing therapy with parents. Their collaboration in producing target settings for treatment programs that involve family values and priorities may decrease the parents’ stress level [31]. The application of MTAO has a great clinical implication, since it is a low intensity program, which can be carried out at home with the follow up of a therapist, reducing economic costs and improving the interaction family—child—therapist.

The aim of the research will be to study the functionality of the affected upper limb in hemiplegia in children aged 6 to 12 years after applying two intervention protocols: MTAO and AOT”.

The hypotheses are: (1) “Both MTAO and AOT improve the functionality of the affected upper limb, with the effect of MTAO being greater” (2) “MTAO increases the spontaneous use and the manual ability in the affected upper limb more than AOT”. (3) “MTAO improves the muscle activity of the affected hand and increases grip strength more than AOT”.

## 2. Materials and Methods

### 2.1. Study Design

A simple-blind (rater) randomized controlled pilot study will be carried out using two groups: AOT (control group) and MTAO (experimental group).

### 2.2. Sample Size

The sample size calculation has been based on the projected effect of the treatment on the main outcome measure, the AHA. To detect an effect size of 1.40 [2], with a significant level of 0.05% and 80% power, a minimum of 10 participants per group will be recruited. To compensate for possible non-responses, 20% more participants will be recruited.

### 2.3. Participants

The participants will be recruited in Spain. The recruitment will be from October 2019 to March 2021. To obtain the sample, a dissemination campaign has been carried out through social networks, the Spanish infantile hemiplegia association Hemiweb and different health professionals involved in the management of these patients, following these inclusion and exclusion criteria.

Inclusion criteria:

- Congenital infantile hemiplegia.
- Aged between 6 and 12 years.
- Lack of use of the affected upper limb.
- Level I–III of the Manual Ability Classification System (MACS). Level I: handles objects easily and successfully; level II: handles most objects but with reduced quality and/or speed of achievement and level III: handles objects with difficulty; needs help to prepare and/or modify activities [32].
- Level I–III in the Gross Motor Function Classification System (GMFCS). Level I: can walk indoors and outdoors and climb stairs without using hands for support, can perform usual activities such as running and jumping and has decreased speed, balance and coordination; level II: can climb stairs with a railing, has difficulty with uneven surfaces, inclined or in crowds of people and has only minimal ability to run or jump and level III: walks with assistive mobility devices indoors and outdoors on level surfaces, may be able to climb stairs using a railing and may propel a manual wheelchair and need assistance for long distances or uneven surfaces [33].

Exclusion criteria:

- Disease not associated with congenital hemiplegia.
- Low cognitive level compatible with attending a special education school.
- Presence of contractures in the affected upper limb affecting the functional movement.
- Surgery in the six months previously to the treatment.
- Botulinum toxin in the two months previously to or during the intervention.
- Pharmacologically uncontrolled epilepsy.

### 2.4. Procedures and Interventions

Patients who meet the inclusion criteria will be referred by email from the recruitment centres to the researcher responsible for the project. An information meeting about the intervention protocols of both therapies will be held at the research centre with the involved families. They will then have to sign an informed consent form. Families who will accept to be included into the research will be trained in the execution of each therapy (AOT or MTAO) by an independent therapist for each therapy, teaching them how they should carry out the activities at home. In addition, they will receive a video with the activities included in the protocol. The treatment will be only initiate when the families and children will be confident about it. A weekly follow-up will be used to avoid any complications and increase the treatment adherence. The families will be requested to fill in a table with the execution time of each proposal activity in the demonstrative videos and how it is performed by the child and the behavior towards it. The follow-up with the families will be conducted online, reviewing all the problems in the activities, modifying them if it is necessary to improve the performance. The family is a key element in improving critical components (intensity, repetition, feedback) of the therapies established with the child [34]. Hadders-Algra et al. [34] state that a family-centred approach creates a richer and more varied range of opportunities by training the family to encourage the children to use the affected upper limb in their usual environment.

Two protocols are designed to be carried out at home, each lasting 20 h, applied during a four-week period (1 h per day from Monday to Friday) at home. The protocols are created based on the previous studies [25,35,36,37]. For the AOT group uni/bimanual activities are designed including 15 sets of daily life upper limb exercises: eight sets for bimanual activities and seven sets for unimanual activities (Table 1).

In the execution, the child will have to watch the video and execute the observed action in the best possible way. The observed action will be repeated for 4 min. All the activities included in the protocol will be observed and imitated during a total time of 60 min without resting periods. During the sessions, each child should sit on a chair with both arms on a table in front of a monitor screen placed 1 m away. The parent will sit on the child’s affected side and should encourage the child by giving verbal suggestions without making any demonstration.

For the MTAO group, the first 15 min will be assigned to the mirror therapy and the remaining 45 min to the action observation therapy. In the first part of the mirror therapy, six activities to be performed with both upper limbs/hands symmetrically have been designed. Even if the affected upper limb cannot successfully complete the proposed action, the child will be asked to do it to the best of his or her ability. (Table 2).

The action observation therapy will be carried out for 45 min (after mirror therapy), using the same activities as in the AOT group (Table 2) and the same positioning of the child, but in this group 3 min are allocated for the visualization and execution of each set without resting during the execution.

Both protocols will include 60-min sessions. The MTAO protocol, begins with 15 min of mirror therapy, followed by 45 min of AOT with a duration of 3 min for each activity, in order to complete 60 min.

The randomization method will be carried out using the Epidat v. 4.2 software (Consellería de Sanidade, Xunta de Galicia, 15703, Spain), which, after a simple randomization process and consecutive sampling, will divide the participants into two groups (TOT and MTAO) with a number of 10 participants per group. A numerical sequence will be obtained which will be kept in opaque sealed envelopes. The envelopes will be opened by a blind researcher, who will decide at random, according to the randomization sequence. Although subject recruitment has begun, due to the Coronavirus pandemic, the trial has been delayed until the follow-up of participants can be ensured.

### 2.5. Outcome Measures

**Figure 1:**
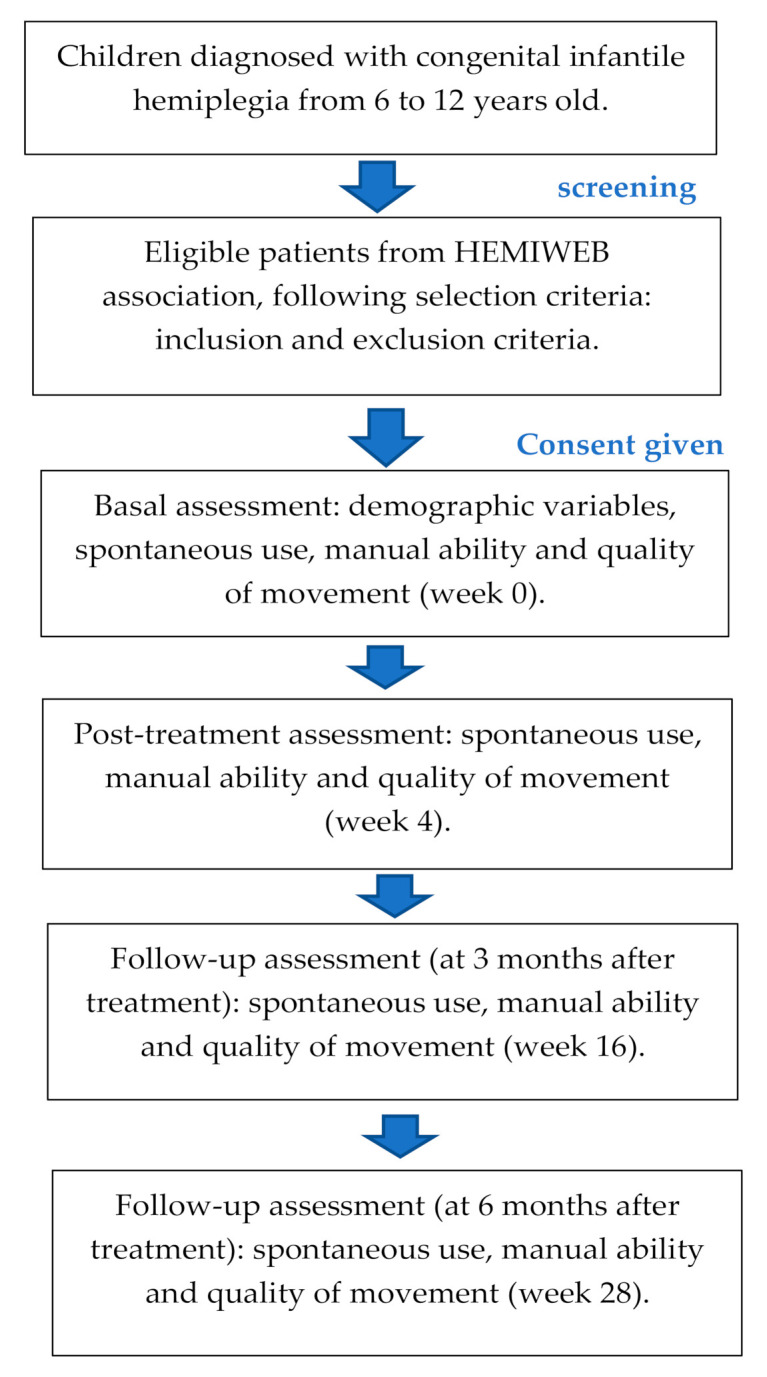
Recruitment, assessment and follow-up.

Four assessments will be performed. The first assessment will be focused on obtaining the data before the treatment and sociodemographic variables (including age and sex), i.e., in week 0 (baseline situation, immediately before starting treatment), whereas the second assessment will be conducted at the end of the treatment, i.e., in week 4 (a total dose of 20 h). Moreover, two follow-up assessments will be done: 3 month and 6 months after treatment in both protocols (Figure 1).

#### 2.5.1. Spontaneous Use

Spontaneous use will be measured using the AHA v. 5.0 scale [38], which is validated for children with infantile hemiplegia and obstetric brachial palsy from 18 months to 12 years. Considering the age range of the participants in this study, the School Kids AHA v. 5.0 Scale will be used, a valid and reliable tool that includes 20 items, with a score from 1 (non-performance) to 4 (effective use) [39,40,41]. The play session will be videotaped and subsequently scored by a trained evaluator and blinded to the group assignment. The Rasch model provides equal interval measures in logits (logarithmic probability units) by converting ordinal rating scale observations into interval levels. In order to facilitate the interpretation of the results, the logit scale is converted into a user-friendly scale from 0 to 100 that continues to be based on the Rasch model and presents interval level data (i.e., AHA) [39].

#### 2.5.2. Manual Ability

Manual ability will be assessed with the JTHFT. This standardized test of simulated functional tasks that quantifies the time to complete a battery of one-handed activities [34]. Activities performed with the paretic hand include turning cards, placing objects, simulating eating, stacking tokens, and manipulating empty and full cans. The JTHFT has shown excellent reliability (0.95–0.99) in children with hand disabilities. [42,43]. The session will be also videotaped and subsequently scored by a trained evaluator and blinded to the group assignment.

#### 2.5.3. Surface Electromyography (EMG) of Extensors and Flexors Muscles of the Wrist

Surface EMG (mDurance Solutions, Granada, Spain) will be used to analyze the muscle recruitment and coordination during maximum isometric contraction. The children will be seat comfortably in a chair. Both shoulders will be at a position of slight flexion and abduction. The elbow will be at flexion, the forearm at neutral position and the hand will be placed naturally on the table. To produce the maximum isometric contraction, children will be asked to grip a cylindrical wood piece. The surface electrodes (36 × 45 mm) will be attached to the skin of the flexor and extensor muscles of the wrist and being parallel to the muscle fibers direction. Previously, the skin will be cleaned using 70% alcohol and fine paper. Three trials of 10 s will be recorded, with a rest of 10 s between trials. Before collected the EMG signals, no contraction signals will be on the screen. The children must avoid intense exercise prior to the test to exclude the effect of residual fatigue. The affected hand will be evaluated first. The root mean square (RMS), integrated EMG (iEMG) and cocotraction ratio (=iEMG of wrist flexors/[iEMG of wrist extensor + iEMG of wrist flexors] × 100) will be calculated and analyzed [14,44,45].

#### 2.5.4. Grasp Strength

In addition, a grip dynamometer (Kern, Balingen, Germany) will be used to assess the grasp strength. The children will be seated in the same position as for the EMG evaluation. The involved hand will be assessed first and then, the non-involved hand. The children will be asked to grasp the dynamometer for 10 s. Three trials will be recorded with a resting interval of 10 s. The grasp dynamometry is a valid and reliable test in cerebral palsy children [46,47].

### 2.6. Data Analysis

Statistical analyses will be performed with SPSS v.24.0 (SPSS Inc., IBM, Chicago, IL, USA). Statistical significance will be set at *p* < 0.05. The test Shapiro-Wilk test will be used to study normality of the sample.

A descriptive analysis of demographic variables will be performed using means and standard deviation for normal distribution variables, and medians and interquartile ranges for non normal distribution variables. The categorial variables will be described using frequencies and %.

To evaluate statistically significant differences intergroup in the variables at baseline, the independent Samples T-test (normal distribution) or the Mann-Whitney U test (non normal distribution) will be used. To study the effects of the interventions on the outcomes measures at four time points, for normal distribution variables, a two-way (intervention × time) repeated measures ANOVA test will be conducted. The Bonferroni correction will be employed for pairwise post-hoc comparisons to further analyze significant interactions. The Greenhouse–Geisser adjustment will be applied to correct for the lack of sphericity (Mauchly’s sphericity test, *p* < 0.05) whenever is necessary. For non-normal distribution variables, the Friedman test will be used. Bivariate correlation coefficients will be estimated to examine the relationship between the subjects’ age, sex, or affected upper limb.

### 2.7. Ethical Aspects

The study was approved (Reference N: 420-20-24) by the CEU-San Pablo university Ethics Committee and will be conducted in accordance to the World Medical Association Declaration of Helsinki. The current Spanish data protection law will be complied with. Before the study began, the consent of the families and children’s caregivers will be provided. The data collected will be used exclusively for this research and anonymisation will be carried out. To ensure the confidentiality and privacy of the data, a numerical code will be assigned to each participant. The outcome measures will be associated with the numerical codes, and not with the personal data of the participants. The identifying list of the numerical codes will be kept by the principal researcher, using a password-protected file. The data of the outcome measures will therefore be anonymised. The principal researcher will be the only person to have access to the entire set of data, which will be kept in password-protected files. In both cases, they will be stored on computers protected by a security password. Under no circumstances will personal information be divulged.

## 3. Discussion

The aim of this research is to compare the effect of two intervention protocols (MTAO and AOT) on the functionality of the affected upper limb in infantile hemiplegia, from 6 to 12 years old.

The motor acts are usually combined to form target-related actions. The same motor acts can be included in actions with different final objectives. It has recently been shown that the visual responses of a subset of the lower parietal and premotor neuron system, studied both during observation and in the execution of grasping acts in different actions (e.g., grasping to place something or grasping to eat), are modulated by the target of the final action (placing or eating) [48,49]. Therefore, during the observation of a person performing an action, the watcher codes the final objective of the observed action (corresponding to the person’s intention) and the objective of the motor act included in that specific action. Thus, the child can internalize the intentionality of the activity observed and the motor act performed. These findings suggest that AOT will increase the spontaneous use of the affected upper limb through observation and imitation of actions, and thus encourage the child’s participation within their natural context and in daily activities. When the proposed activities are targeted at a functional goal or represent everyday activities, this automatic use is encouraged within the everyday environment. Considering the reduction of selective movements and the difficulty of executing adequate movements, mirror therapy could help to improve movement quality in dynamics and manual dexterity measured through grasping [37]. In our study, improvements in the coordination of wrist flexor and extensor contraction and more fluid manual dexterity can be obtained. Therefore, the MTAO protocol could induce changes in the spontaneous use and in the quality of movement of the affected upper limb.

This research has some limitations. Firstly, the lack of cross-cultural validation in Spanish of some of the measurement tools used, such as the AHA. In addition, a group with conventional intervention has not been included. As strengths, it highlights that it is the first research to evaluate the effect of mirror therapy combined with action observation therapy on upper limb functionality. In addition, the intervention protocols will be carried out at home, which means reducing economic costs and optimizing family resources. Finally, the results will improve clinical practice.

The results of our randomized controlled trial will be disseminated to the scientific community through scientific (peer-reviewed) journals and congress. In addition, research results will be presented to stakeholders, including patients, families and physicians.

## 4. Conclusions

The study of functional changes in the affected upper extremity in children with hemiplegia after the use of AOT or MTAO will help clinicians in the design and selection of appropriate therapeutic strategies to improve the functionality of the affected upper extremity. In addition, this will allow the home-based implementation of the interventions with the involvement of the family.

## Figures and Tables

**Table 1:** Activities for Action Observation therapy.

**Unimanual activities**	Remove the lid from a container, remove marbles and place them in a glass. Pour water into a glass. Take a colored card, turn it over and place it with a similar figure to make pairs. Use a stamp, making figures in a horizontal plane, that is, on a paper on the table. Take coins from the table, put them in a slotted container. Take a sponge and dip it in finger paint, and then squeeze the foam in a horizontal plane, on the table. Lift the open lid of a tube containing colored paint and squish it to throw the paint onto a sheet of paper and spread it out. Use a toy fishing rod and catch magnetic animals or just catch magnetic animals.
**Bimanual Activities**	Chop up tissue paper. Wet a small towel and drain it. Stick cards on your sign on the table on a horizontal plane and/or on felt paper on a vertical plane. Make a large ball of clay and roll it with both hands until it is thrown at the end of the table. Open a coin box and take out 5 coins and put them back in and close the coin box. Build figures with Lego pieces. Apply glue stick on a piece of paper and throw the pieces of tissue paper cut earlier with both hands and glue them to create a picture.

**Table 2:** Activities for mirror therapy into MTAO group.

**Activities**	Touch with the tips of the fingers a plasticine base for 2 min (Rest 30 s) The movement of the fingers is worked; the thumb touches each one of the fingertips for 2 min. (Rest 30 s) We work the thumb and index finger clamp with the contact of both fingers putting a resistance through a rubber between thumb and index finger for 2 min. (Rest 30 s) We will place a sponge in each of the hands to work the global grip. Squeeze and release the object and therefore also the grip strength for 2 min. (Rest 30 s) Extension movement of both wrists on a plasticine base for 2 min. (Rest 30 s) Prono-supination of the forearm, starting from a pronation position for 2 min. (Rest 30 s before starting TOA)

## References

[1] Monge Pereira E., Molina Rueda F., Alguacil Diego I.M., de la Cuerda R.C., de Mauro A., Miangolarra Page J.C. (2014). Empleo de sistemas de realidad virtual como método de propiocepción en parálisis cerebral: Guía de práctica clínica. Neurologia.

[2] Sgandurra G., Ferrari A., Cossu G., Guzzetta A., Biagi L., Tosetti M., Fogassi L., Cioni G. (2011). Upper limb children action-observation training (UP-CAT): A randomised controlled trial in Hemiplegic Cerebral Palsy. BMC Neurol..

[3] Riddell M., Kuo H.C., Zewdie E., Kirton A. (2019). Mirror movements in children with unilateral cerebral palsy due to perinatal stroke: Clinical correlates of plasticity reorganization. Dev. Med. Child Neurol..

[4] Vosberg D.E., Beaulé V., Torres-Berrío A., Cooke D., Chalupa A., Jaworska N., Cox S.M.L., Larcher K., Zhang Y., Allard D. (2019). Neural function in DCC mutation carriers with and without mirror movements. Ann. Neurol..

[5] Ejaz N., Xu J., Branscheidt M., Hertler B., Schambra H., Widmer M., Faria A.V., Harran M.D., Cortes J.C., Kim N. (2018). Evidence for a subcortical origin of mirror movements after stroke: A longitudinal study. Brain.

[6] Eliasson A.C., Forssberg H., Hung Y.C., Gordon A.M. (2006). Development of hand function and precision grip control in individuals with cerebral palsy: A 13-year follow-up study. Pediatrics.

[7] Boyd R., Sakzewski L., Ziviani J., Abbott D.F., Badawy R., Gilmore R., Provan K., Tournier J.D., Macdonell R.A., Jackson G.D. (2010). INCITE: A randomised trial comparing constraint induced movement therapy and bimanual training in children with congenital hemiplegia. BMC Neurol..

[8] Palomo-Carrión R., Romero-Galisteo R.-P., Pinero-Pinto E., López-Muñoz P., Romay-Barrero H., José F.-M. (2020). Application of Low-Intensity Modified Constraint-Induced Movement Therapy to Improve the Affected Upper Limb Functionality in Infantile Hemiplegia with Moderate Manual Ability: Case Series. Children.

[9] Palomo-Carrión R., Pinero-Pinto E., Ando-LaFuente S., Ferri-Morales A., Bravo-Esteban E., Romay-Barrero H. (2020). Unimanual Intensive Therapy with or without Unaffected Hand Containment in Children with Hemiplegia. A Randomized Controlled Pilot Study. J. Clin. Med..

[10] Pandyan A.D., Gregoric M., Barnes M.P., Wood D., Van Wijck F., Burridge J., Hermens H., Johnson G.R. (2005). Spasticity: Clinical perceptions, neurological realities and meaningful measurement. Disabil. Rehabil..

[11] Nielsen J.B., Crone C., Hultborn H. (2007). The spinal pathophysiology of spasticity—From a basic science point of view. Acta Physiol..

[12] Bleyenheuft Y., Gordon A.M. (2013). Precision grip control, sensory impairments and their interactions in children with hemiplegic cerebral palsy: A systematic review. Res. Dev. Disabil..

[13] Sarcher A., Raison M., Ballaz L., Lemay M., Leboeuf F., Trudel K., Mathieu P.A. (2015). Impact of muscle activation on ranges of motion during active elbow movement in children with spastic hemiplegic cerebral palsy. Clin. Biomech..

[14] Xu K., Mai J., He L., Yan X., Chen Y. (2015). Surface electromyography of wrist flexors and extensors in children with hemiplegic cerebral palsy. PM R.

[15] Houwink A., Aarts P.B., Geurts A.C., Steenbergen B. (2011). A neurocognitive perspective on developmental disregard in children with hemiplegic cerebral palsy. Res. Dev. Disabil..

[16] Taub E., Wolf S.L. (1997). Constraint-induced (CI) movement techniques to facilitate upper extremity use in sotrke patients. Top. Stroke Rehabil..

[17] Sunderland A., Tuke A. (2005). Neuroplasticity, learning and recovery after stroke: A critical evaluation of constraintinduced therapy. Neuropsychol. Rehabil..

[18] Chung C.Y., Chen C.L., Wong A.M. (2011). Pharmacotherapy of spasticity in children with cerebral palsy. J. Formos. Med. Assoc..

[19] Pollock A., Farmer S.E., Brady M.C., Langhorne P., Mead G.E., Mehrholz J., van Wijck F. (2014). Interventions for improving upper limb function after stroke. Cochrane Database Syst. Rev..

[20] Ramachandran V.S., Rogers-Ramachandran D., Cobb S. (1995). Touching the phantom limb. Nature.

[21] Deconinck F.J., Smorenburg A.R., Benham A., Ledebt A., Feltham M.G., Savelsbergh G.J. (2015). Reflections on mirror therapy: A systematic review of the effect of mirror visual feedback on the brain. Neurorehabilit. Neural Repair.

[22] French B., Thomas L.H., Coupe J., McMahon N.E., Connell L., Harrison J., Sutton C.J., Tishkovskaya S., Watkins C.L. (2016). Repetitive task training for improving functional ability after stroke. Cochrane Database Syst. Rev..

[23] Bruchez R., Jequier Gygax M., Roches S., Fluss J., Jacquier D., Ballabeni P., Grunt S., Newman C.J. (2016). Mirror therapy in children with hemiparesis: A randomized observer-blinded trial. Dev. Med. Child Neurol..

[24] Stinear C.M., Barber P.A., Coxon J.P., Fleming M.K., Byblow W.D. (2008). Priming the motor system enhances the effects of upper limb therapy in chronic stroke. Brain.

[25] Yeves-Lite A., Zuil-Escobar J.C., Martínez-Cepa C., Romay-Barrero H., Ferri-Morales A., Palomo-Carrión R. (2020). Conventional and Virtual Reality Mirror Therapies in Upper Obstetric Brachial Palsy: A Randomized Pilot Study. J. Clin. Med..

[26] Feltham M.G., Ledebt A., Deconinck F.J., Savelsbergh G.J. (2010). Mirror visual feedback induces lower neuromuscular activity in children with spastic hemiparetic cerebral palsy. Res. Dev. Disabil..

[27] Ramachandran V.S., Altschuler E.L. (2009). The use of visual feedback, in particular mirror visual feedback, in restoring brain function. Brain.

[28] Buccino G., Arisi D., Gough P., Aprile D., Ferri C., Serotti L., Tiberti A., Fazzi E. (2012). Improving upper limb motor functions through action observation treatment: Pilot study in children with cerebral palsy. Dev. Med. Child Neurol..

[29] Smith T.B., Oliver M.N., Innocenti M.S. (2001). Parenting stress in families of children with disabilities. Am. J. Orthopsychiatry.

[30] McConachie H., Colver A.F., Forsyth R.J., Jarvis S.N., Parkinson K.N. (2006). Participation of disabled children: How should it be characterised and measured?. Disabil. Rehabil..

[31] Raina P., O’Donnell M., Rosenbaum P., Brehaut J., Walter S.D., Russell D., Swinton M., Zhu B., Wood E. (2005). The health and well-being of caregivers of children with cerebral palsy. Pediatrics.

[32] Eliasson A.C., Krumlinde-Sundholm L., Rösblad B., Beckung E., Arner M., Ohrvall A.M., Rosenbaum P. (2006). The Manual Ability Classification System (MACS) for children with cerebral palsy: Scale development and evidence of validity and reliability. Dev. Med. Child Neurol..

[33] Palisano R., Rosenbaum P., Walter S., Russell D., Wood E., Galuppi B. (1997). Development and reliability of a system to classify gross motor function in children with cerebral palsy. Dev. Med. Child Neurol..

[34] Hadders-Algra M., Boxum A.G., Hielkema T., Hamer E.G. (2017). Effect of early intervention in infants at very high risk of cerebral palsy: A systematic review. Dev. Med. Child Neurol..

[35] Sgandurra G., Ferrari A., Cossu G., Guzzetta A., Fogassi L., Cioni G. (2013). Randomized trial of observation and execution of upper extremity actions versus action alone in children with unilateral cerebral palsy. Neurorehabil. Neural Repair.

[36] Beani E., Menici V., Ferrari A., Cioni G., Sgandurra G. (2020). Feasibility of a Home-Based Action Observation Training for Children With Unilateral Cerebral Palsy: An Explorative Study. Front Neurol..

[37] Gygax M.J., Schneider P., Newman C.J. (2011). Mirror therapy in children with hemiplegia: A pilot study. Dev. Med. Child Neurol..

[38] Holmefur M.M., Krumlinde-Sundholm L. (2016). Psychometric properties of a revised version of the Assisting Hand Assessment (Kids-AHA 5.0). Dev. Med. Child Neurol..

[39] Krumlinde-Sundholm L., Eliasson A.C. (2003). Development of the Assisting Hand Assessment: A Rasch-built measure intended for children with unilateral upper limb impairments. Scand. J. Occup. Ther..

[40] Holmefur M., Aarts P., Hoare B., Krumlinde-Sundholm L. (2009). Test-retest and alternate forms reliability of the assisting hand assessment. J. Rehabil. Med..

[41] Krumlinde-Sundholm L., Holmefur M., Kottorp A., Eliasson A.C. (2007). The assisting hand assessment: Current evidence of validity, reliability, and responsiveness to change. Dev. Med. Child Neurol..

[42] Jebsen R.H., Taylor N., Trieschmann R.B., Trotter M.J., Howard L.A. (1969). An objective and standardized test of hand function. Arch. Phys. Med. Rehabil..

[43] Taylor N., Sand P.L., Jebsen R.H. (1973). Evaluation of hand function in children. Arch. Phys. Med. Rehabil..

[44] Schmidt-Rohlfing B., Bergamo F., Williams S., Erli H.J., Rau G., Niethard F.U., Disselhorst-Klug C. (2006). Interpretation of surface EMGs in children with cerebral palsy: An initial study using a fuzzy expert system. J. Orthop. Res..

[45] Xu K., He L., Mai J., Yan X., Chen Y. (2015). Muscle recruitment and coordination following constraing-induced movement therapy with electrical stimulation on children with hemiplegic cerebral palsy: A randomized controlled trial. PLoS ONE.

[46] Dekkers K., Smeets R., Janssen-Potten Y., Gordon A.M., Speth L.A., Rameckers E.A. (2019). Psychometric evaluation of 2 new upper extremity funciontal strength tests in children with cerebral palsy. Phys. Ther..

[47] Dekkers K., Janseen-Potten Y., Gordon A.M., Speth L., Smeets R., Rameckers E. (2019). Reliability of máximum isometric arm, grip and pinch strength measurements in children (7–12 years) with unilateral spastic cerebral palsy. Disabil. Rehabil..

[48] Bonini L., Rozzi S., Serventi F., Simone L., Ferrari P.F., Fogassi L. (2010). Ventral premotor and inferior parietal cortices make distinct contribution to action organization and intention understanding. Cereb. Cortex.

[49] Fogassi L., Ferrari P.F., Gesierich B., Rozzi S., Chersi F., Rizzolatti G. (2005). Parietal lobe: From action organization to intention understanding. Science.

